# Effect of Paracetamol, Dexketoprofen Trometamol, Lidocaine Spray, and Paracervical Block Application for Pain Relief during Suction Termination of First-Trimester Pregnancy

**DOI:** 10.1155/2013/869275

**Published:** 2013-12-24

**Authors:** Gökhan Açmaz, Hüseyin Aksoy, Nil Özoğlu, Ülkü Aksoy, Evrim Albayrak

**Affiliations:** ^1^Department of Obstetrics and Gynecology, Kayseri Education and Research Hospital of Medicine, 38210 Kayseri, Turkey; ^2^Department of Obstetrics and Gynecology, Kayseri Military Hospital, Kayseri, Turkey; ^3^Clinic of Family Planning, Kayseri Education and Research Hospital of Medicine, 38210 Kayseri, Turkey; ^4^Department of Obstetrics and Gynecology, Kayseri Education and Research Hospital of Medicine, Kayseri, Turkey; ^5^Department of Midwifery, Erciyes University School of Medicine, 38210 Kayseri, Turkey

## Abstract

The aim of the study was to investigate the analgesic efficacy of preoperative oral dexketoprofen trometamol, intravenous paracetamol, lidocaine spray, and paracervical block with ultracaine on curettage procedure. A total of 111 subjects with the request of pregnancy termination between 5 and 7 weeks of gestation were included in the study. The first group (control group) consisted of 20 participants without medication. The second group consisted of 25 participants receiving 2 puffs of lidocaine sprays on cervical mucosa. The third group consisted of 20 participants receiving oral 25 mg dexketoprofen trometamol. The fourth group consisted of 23 participants receiving 1000 mg intravenous paracetamol and the fifth group consisted of 23 participants receiving paracervical block with ultracaine. Paracervical block reduced pain score significantly in both intraoperative and postoperative periods. All analgesic procedures were significantly effective for reducing pain in postoperative period. Paracervical block may be the best method for reducing pain scores in intraoperative and postoperative periods during curettage procedure. All analgesic procedures such as lidocaine, paracetamol, ultracaine, and paracervical block with ultracaine can be used for reducing pain score in postoperative period. This trial is registered with NCT01947205.

## 1. Introduction

Curettage, a surgical procedure, is commonly used for the first-trimester pregnancy termination. Patients frequently experience pain from moderate to severe during gynecologic procedures that involve the uterine cavity [1]. Clinicians have a tendency to use local anesthesia. A study showed that only 10% of clinics use general anesthesia, so local anesthesia becomes a dominant method with 58% use in clinics and 32% use of intravenous sedation with local anesthesia [2].

Also known as “paracetamol,” acetaminophen (N-acetyl-4-aminophenol), which has not only analgesic but also antipyretic properties and has been widely used as an active ingredient in many approved drugs, is one of the most widely used medicines in the world [3]. It has been shown that paracetamol can be used after gynecologic surgical procedures [4]. Nonsteroidal anti-inflammatory drugs (NSAIDs) show their effect by cyclooxygenase inhibition and do not discriminate two enzymes cyclooxygenase (COX)-1 and -2. The reduced activity of COX-1 is associated with side effects of NSAIDs as gastrointestinal bleeding and platelet dysfunction. Dexketoprofen trometamol, the active enantiomer of racemic ketoprofen, is classified as NSAID with analgesic and antipyretic properties [5]. Api et al. showed that curettage-associated pain may be reduced by administration of oral dexketoprofen during fractional curettage [6].

It is revealed that cervical dilation, contraction, or cramping pain is transmitted by sympathetic fibers, which travel in the uterosacral ligament and insert into the cervix. Therefore, paracervical injections may be best for preventing this cramping pain. Moreover prior studies showed that paracervical block can be used as an anesthetic procedure for suction curettage [7]. Lidocaine spray, which shows its effect by reduction of generation and conduction of peripheral pain impulses in dysfunctional or damaged nociceptors situated directly below the application site, is reported to produce significant pain relief even with the absence of clinically significant serum levels [8]. The metered-dose lidocaine pump spray can be used for anesthetizing mucosal membranes. Lidocaine has been reported to provide effective analgesia on surgical wounds [9].

We conducted a prospective, randomized, placebo-controlled trial to investigate the analgesic efficacy of preoperative oral dexketoprofen trometamol (Dexofen 25 mg tb Atabay Ilaç Turkey), intravenous paracetamol (Parol 1 mg Atabay Ilaç Turkey), lidocaine spray (Xylocaine Pump 100 mg 50 mL Sprey Astra Zeneca), and paracervical block with ultracaine on curettage procedure.

## 2. Methods

A randomized, double-blind, placebo controlled trial was designed at our family planning clinics between November 2012 and August 2013. The study protocol was approved by the ethics committee of Erciyes University. Study and control groups consisted of pregnant multiparous participants between 35 and 48 days of gestation (5 to 7 weeks of gestation) with a single viable intrauterine pregnancy requesting termination of pregnancy. These participants were scheduled for performing suction curettage and none of the participants received medication such as analgesics and misoprostol, up to 7 days.

Exclusion criteria were incomplete abortions, diabetes mellitus, tendency to bleed such as thrombocytopenia, factor deficiency and functional disorders of platelets, pelvic infection, known cervical stenosis, significantly impaired respiratory or cardiac conduction functions, active liver disease, renal disease, previous adverse reaction to any of the drugs used in the study, and patients who are unable to understand how to score a 10 cm visual analog scale (VAS) pain score. Moreover, patients who described chronic pelvic pain prior to the study or patients who rated their pain level on a continuous 100 mm VAS different from 0 (no pain) just before the study were not included in the study.

Previously published studies for assessing pain scores during minor gynecologic surgical procedures illustrated that at least a 2.73 cm difference between the pain scores could be regarded as a clinically significant difference [10]. It was estimated that at least 18 subjects were required in each arm to detect a difference between the 2 groups at least 2.73 cm on a 10 cm VAS scale when assuming a power of 80% at a type I error of 0.05 and SD of 2.7 cm.

After providing their informed consent, a total of 111 subjects with request of pregnancy termination between 5 and 7 weeks of gestation were randomly assigned into 1 of 5 study groups. Subjects received 25 mg of oral dexketoprofen trometamol, 1000 mg IV paracetamol, two-puff xylocaine administration on cervical surface, paracervical block with ultracaine, or similar-appearing placebo drugs by using a computer-generated random number chart (SPSS, version 20.0 for Windows; SPSS, Inc, Chicago, IL) before the study. The first group (control group) consisted of 20 participants and they did not receive any treatment. In this group, 5 of the patients received placebo tablet, 5 received saline solution, 5 received paracervical isotonic solution, and 5 received puff saline solution. The second group consisted of 25 participants receiving lidocaine spray on cervical mucosa. The third group consisted of 20 participants receiving oral dexketoprofen trometamol. The fourth group consisted of 23 participants receiving 1000 mg intravenous paracetamol and the fifth group consisted of 23 participants receiving paracervical block with ultracaine. In all, 111 consecutive patients' numbers were written on envelopes, while the assignment codes were written on separate papers that were put into the consecutively numbered yellow opaque envelopes, which were then pasted. During curettage, the envelope with the patient number on its cover was opened to reveal the randomization by the responsible nurse and she prepared the trial medications accordingly.

The patients, the anesthetist performing VAS, and the gynecologist performing the procedure were blinded to the contents of the oral, intravenous, paracervical, and puff medications. The oral, intravenous medications were administered 30 minutes before the suction curettage due to the pharmacokinetic properties of dexketoprofen, paracetamol, since they are known to possess a very rapid onset of action (within 30 minutes of administration) with a long-lasting effect (at least for 6–8 hours). The suction curettage was performed by the same gynecologist to maintain consistency and limit confounding variables. We administered 2 puffs of lidocaine spray on cervical surface and waited for three minutes to allow the anesthetic to take effect.

The procedure and paracervical block were performed according to our clinics description as the following: a sterile bivalve speculum was introduced into the vagina, then cervix and vagina were washed with antiseptic solution. The cervix was grasped with tenaculum and straightened according to the uterine axis. Paracervical block was achieved using a 27-gauge spinal needle. Ultracaine was injected at the tenaculum site, and the remainder was distributed equally around the cervicovaginal junction at 5 and 7 o'clock.

The suction curettage was accomplished through the following steps: the cervix was dilated to Hegar number 5 to 6 (Aesculap, Ag and Co. KG, Tuttlingen, Germany) if necessary; the uterine depth was measured using a hysterometry; and then a 60 mL syringe with a self-locking mechanism of the plunge was used for performing suction curettage.

The patients were observed for 30 minutes after the curettage procedure. No further followup was scheduled. Patients were asked to rate their pain level on a continuous 100 mm VAS from 0 (no pain) to 10 (the worst pain ever). Pain scoring was performed at 3 different time points: prior to the procedure (*t*1), during the procedure (immediately following the removal of the speculum from the vagina at the end of the suction curettage, the patients were asked to score their pain level experienced during the procedure) (*t*2), and 30 minutes after the procedure (*t*3). We documented the patients' demographics: age, parity, weight, and height.

### 2.1. Statistics

To test the normality assumption of the data, Shapiro-Wilk and Kolmogorov-Smirnov were used, respectively. Variance homogeneity assumption was tested with Levene test for the variables age, BMI, and gravida. Values are expressed as mean ± standard deviation or median (25th percentile–75th percentile). One-way analysis of variance (ANOVA) test or Kruskal-Wallis *H* test was performed for the comparison of differences between groups. All homogeneity of variance tests and normality tests was accepted for the variables age, BMI, and gravida. Tukey HSD post hoc tests were used for the multiple comparisons for the variables of age, BMI, and gravida. Other comparisons were made with the nonparametric test (Kruskal-Wallis *H* test). All analyses were performed using IBM SPSS Statistics 20.0 (SPSS IBM, Inc., Chicago, IL) and *P* < 0.05 was considered statistically significant.

## 3. Results 

Patients who described chronic pelvic pain prior to the study or patients who rated their pain level on a continuous 100 mm VAS different from 0 (no pain) just before the study were not included in the study. All participants reported their pain level 0 on a continuous 100 mm VAS prior to the study. General characteristics and pain scores of both control and study groups are illustrated in Table 1.

Paracervical block was the best choice for reducing pain during curettage procedure (*t*2: intraoperative). All analgesic procedures were significantly effective in reducing pain in postoperative period. Significant pain reduction was achieved for both intraoperative and postoperative periods by using paracervical bloc.

Both side effects of the medications and analgesic costs for each patient are shown in Table 2.

Total complication rates were 1 (5%), 1 (4%), 3 (15%), 2 (8.69%), and 2 (8.69%) in control, lidocaine spray, dexketoprofen, paracetamol, and paracervical block group, respectively. The cheapest method was lidocaine spray for postoperative analgesia, however lidocaine did not reduce pain scores effectively during intraoperative period.

## 4. Discussion

Blumenthal and Remsburg showed that suction curettage might be performed on an outpatient basis under local anesthesia and cost of patients might be reduced by using suction curettage practice on an outpatient basis [11]. Following on from this study many clinics intend to carry out suction curettage as an outpatient procedure; however, the best choice for analgesic remains a debate. Therefore, this study is aimed at defining the most effective anesthetic method in patients undergoing curettage in outpatient settings. During cervical dilation, pain signals are carried by parasympathetic fibers that accompany the uterine vessels and cardinal ligament [12]. In addition to pain with cervical dilation, contraction or cramping pain is transmitted by sympathetic fibers from the ovarian plexus and inferior hypogastric nerve, which travels in the uterosacral ligament and inserts into the cervix at the 5 and 7 o'clock positions [7].

Paracetamol, a nonopioid agent, is believed to primarily act upon the central nervous system by way of central cyclooxygenase inhibition and probably has an indirect influence on the serotoninergic system. Paracetamol has a good safety profile and easily passes through the brain barrier, which assures it as an effective analgesic [13]. Api et al. conducted a double-blind, randomized, placebo-controlled trial with paracetamol and investigated analgesic efficacy of 1 g intravenous paracetamol during fractional curettage. They concluded that there was no significant difference in pain scores of patients undergoing fractional curettage when the use of i.v. paracetamol was compared with placebo [10]. In another study, intravenous (i.v.) paracetamol 1 g preoperatively or intraoperatively was administered to hysterectomy patients and its postoperative analgesic effects were assessed. It was concluded that preemptive i.v. paracetamol 1 g provided good quality postoperative analgesia with decreased consumption of morphine and minimal side effects [14]. Our results were similar to the ones in the literature. There was no significant difference between pain scores of paracetamol and control group during suction curettage; however, pain score significantly reduced by using paracetamol in postoperative period.

Lidocaine spray (Xylocaine Pump 100 mg 50 mL Sprey Astra Zeneca) is a simple and convenient local anesthetic agent with minimal adverse effect. However, there is limited evidence regarding the effectiveness of its use in pain control during suction termination of pregnancy [15]. Paracervical or intrauterine application of lidocaine during curettage is common in literature, whereas efficacy of lidocaine in spray form on curettage is not well known.

Xu et al. claimed that dicaine-containing lubricant jelly for suction termination of first-trimester pregnancy had no effect on pain scores. However, the same study reported significantly more satisfactory cervical dilatation in the group using dicaine-containing lubricant jelly compared with the group using lignocaine injection [16]. Conversely Li et al. demonstrated that lidocaine gel significantly reduced pain scores during curettage compared with the placebo group, but they concluded with subgroup analysis that lignocaine gel reduced overall intraoperative pain only in the multiparous but not the nulliparous subjects. The reason for the differential effect of parity was uncertain [15]. In the study of Lis, both control and study groups received premedication with misoprostol, diazepam, and pethidine. We agree with the opinion that these medications make participants decisions questionable. Theoretically, topical anaesthetic application could only reduce the pain component because of cervical manipulation/dilatation but not the pain component because of uterine instrumentation and contraction, because the upper part of the uterus derives its sensory innervation differently from the cervix (the upper part of the uterus is innervated by the ovarian nerve plexus, whereas the lower part and the cervix are innervated by the parasympathetic plexus S2–S4). Although our participants in study group revealed low pain score in lidocaine group, there was no significant difference between the groups.

Prior studies showed that paracervical block can be used as an anesthetic procedure for suction curettage [7]. Ovolabi evaluated and compared paracervical block with diclofenac for pain relief during suction curettage for surgical termination of pregnancy. The local anaesthetic infiltration of the cervix in combination with diclofenac provides better pain relief than diclofenac alone during and after the suction curettage [17]. Conversely, Api et al. concluded that administration of intrauterine lidocaine or oral dexketoprofen appears to be effective in relieving fractional curettage-associated pain. However, a combination of them does not work better in further reduction of pain [6].

Our results showed that paracervical block provides better pain relief than dexketoprofen. Although paracervical block provided significant pain relief during surgical procedure and postoperative period, there are some concerns among authors regarding the use of paracervical block. It can be an insufficient anesthetic method for curettage because pain signals of uterine fundus are carried by different neurons compared to cervix and paracervical block which has well-known adverse effects, which range from mild toxicity such as numbness around the mouth and dizziness to convulsion and respiratory arrest. Five deaths were reported due to lidocaine use to induce paracervical anesthesia [18].

Lidocaine, paracetamol, and dexketoprofen can be accepted as relatively less invasive analgesic methods than paracervical block. However, in order to provide an effective analgesia during intraoperative period, at least paracervical block should be used. This situation requires choosing a reliable, innocent, and effective anesthetic method in patients undergoing curettage in outpatient settings; therefore, there is a clear need for well-designed, large-scale, prospective observational studies in the field of curettage and pain.

## Figures and Tables

**Table 1:** General characteristics and pain scores of groups.

	Control (*n* = 20)	Lidocaine spray (*n* = 25)	Dexketoprofen (*n* = 20)	Paracetamol (*n* = 23)	Ultracaine (*n* = 23)	*P* value
Age	31.45 ± 7.67	32.08 ± 6.92	30.85 ± 6.60	33.61 ± 7.10	32.04 ± 6.21	0.749
BMI	26.75 ± 5.52	27.84 ± 5.91	27.50 ± 6.13	25.87 ± 5.28	29.26 ± 6.49	0.381
Gravida	5 ± 2.25	4.2 ± 1.41	4.1 ± 1.17	4.70 ± 2.27	4.39 ± 1.95	0.508
Parity	2.5 (2–3.75)	2 (2-3)	2 (2-3)	3 (2–4)	2 (2–5)	0.745
Gestational week	6 (5–7)	5 (5–6.5)	5 (5–6.75)	6 (5–7)	6 (5–6)	0.900
VAS (intra-op)	8 (6.25–9)^a^	6 (5–8)^a^	7.5 (5–8)^a^	7 (4–8)^a^	5 (3–5)^b^	<0.001
VAS (post-op)	4 (2.25–5.75)^a^	2 (1–3)^b^	2 (2-3)^b^	2 (1–3)^b^	2 (1–4)^b^	0.001

Values are expressed as mean ± standard deviation or median (25th percentile–75th percentile). The variables were compared with independent *t*-test and Kruskal-Wallis *H* test. Groups with different superscript letters were found to have statistically significant differences.

**Table 2:** Analgesic costs and side effects of medications.

	Control (*n* = 20)	Lidocaine spray (*n* = 25)	Dexketoprofen (*n* = 20)	Paracetamol (*n* = 23)	Ultracaine (*n* = 23)
Nausea	1 (5)	1 (4)	1 (5)	0 (0)	2 (8.69)
Vomiting	0 (0)	0 (0)	0 (0)	0 (0)	1 (4.34)
Rash	0 (0)	0 (0)	0 (0)	1 (4.34)	0 (0)
Stomach complaints	0 (0)	0 (0)	3 (15)	1 (4.34)	0 (0)
Bradycardia	1 (5)	0 (0)	0 (0)	0 (0)	2 (8.69)

Total complication rate	1 (5)	1 (4)	3 (15)	2 (8.69)	2 (8.69)
Analgesic cost for each patient	0$	0.02$	0.18$	2.47$	1.02$

Values were expressed as *n* (%) or dollars.
